# A Review of NIST Primary Activity Standards for ^18^F: 1982 to 2013

**DOI:** 10.6028/jres.119.013

**Published:** 2014-08-27

**Authors:** Denis E Bergeron, Jeffrey T Cessna, Bert M Coursey, Ryan Fitzgerald, Brian E Zimmerman

**Affiliations:** National Institute of Standards and Technology, Gaithersburg, MD 20899

**Keywords:** anticoincidence, discrepancy, F-18, ionization chamber, liquid scintillation counting, primary standard

## Abstract

The new NIST activity standardization for ^18^F, described in 2014 in Applied Radiation and Isotopes (v. 85, p. 77), differs from results obtained between 1998 and 2008 by 4 %. The new results are considered to be very reliable; they are based on a battery of robust primary measurement techniques and bring the NIST standard into accord with other national metrology institutes. This paper reviews all ten ^18^F activity standardizations performed at NIST from 1982 to 2013, with a focus on experimental variables that might account for discrepancies. We have identified many possible sources of measurement bias and eliminated most of them, but we have not adequately accounted for the 1998–2008 results.

## 1. Introduction

**Fig. 1:**
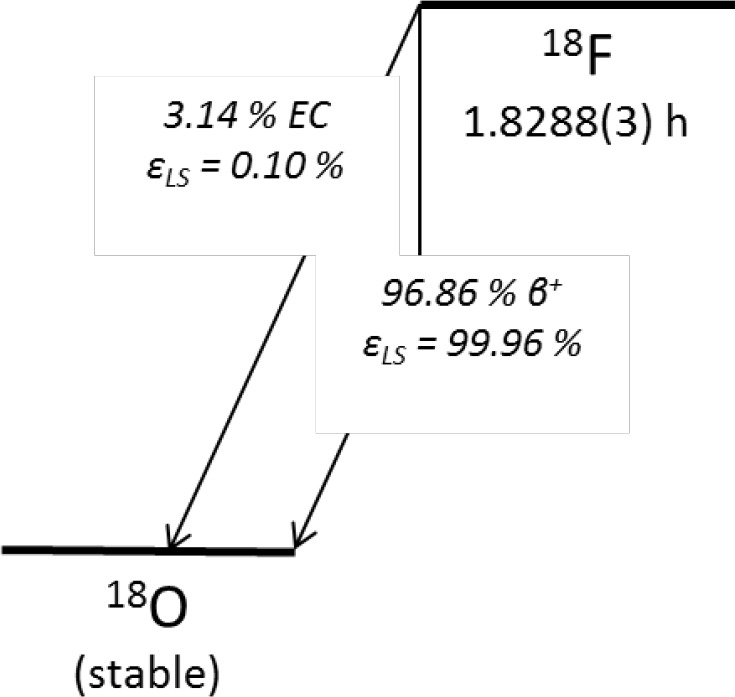
Decay scheme for ^18^F, with LS efficiencies for each branch as calculated by MICELLE2. The calculations were for a Hionic Fluor cocktail with 10 % added water. The CIEMAT/NIST output (H3X.TAB) at *ε*_H-3_ = 0.50 is shown.

F-18 decays primarily by positron emission to ^18^O with a half-life of 1.8288(3) h [1]—see Fig. 1. Its use, as part of the molecule 2-[^18^F]Fluoro-2-Deoxy-D-Glucose (FDG), as a positron emission tomography (PET) agent has revolutionized medical imaging [2], complementing the structural information attainable using x-ray-based imaging with invaluable functional details [3]. PET is most typically associated with oncology, where the accumulation of FDG in metabolically active tumors allows for the highly specific detection of small lesions and metastases. However, the technique has captured the imagination (quite literally, one could argue) with its promise of insight into the physiological function of the brain. Alongside functional magnetic resonance imaging (fMRI), PET imaging has revealed in remarkable detail the physiological bases for vast swaths of human mental processes [4–7]. More recently, PET has emerged as a technique with an important role to play in safeguarding those processes. Researchers are learning to distinguish between different forms of dementia—sometimes enabling the selection of different, mutually exclusive, interventions—using PET [8–10]. Moreover, the “inherently quantitative” nature of PET and its sensitivity to cardiovascular and neurological diseases, specific gene expression, and cancerous lesions are being increasingly utilized in drug trials [11–13]. Whether to map the metabolic pathway of a novel pharmaceutical through labeling experiments or to track disease progression or response to therapy in a quantitative (and thus comparable between different clinical sites and at different times) manner, PET is playing a growing role in pharmaceutical development and approval.

The main impact of ^18^F, then, is on the quality and duration of peoples’ lives. Some readers might also find economic considerations compelling. In 2011, 1.85 million PET and PET/CT exams were performed in the United States, an increase of 6 % over the 1.74 million exams prerformed in 2010 [14,15]. In 2012, the average hospital charge to a patient for a PET exam was $3 000.00 for an estimated $5.5 billion total annual U.S. expenditure on PET exams [16]. The U.S. PET systems market in 2010 was valued at $324 million [17].

The extant medical infrastructure and the general ubiquity evident from the economic data make [^18^F]FDG-PET a logical starting point for industry-wide standardization for absolute quantitation in molecular imaging [18]. Absolute image quantitation is a critical part of what appears to be the emerging paradigm in 21^st^ century drug development and approval [19,20]. The various sources of uncertainty, both physical and biological, in obtaining quantitative data from clinical PET images have been reviewed in the literature [21]. The first step in any quantitative measurement using a medical scanner is calibration. For absolute quantitation in terms of Bq·mL^−1^, it is desirable that the calibration be traceable to some measurement standard.

Primary activity standards for ^18^F have been independently established by many national metrology institutes (NMIs) [22–36], including the National Institute of Standards and Technology (NIST) [25,37–39]. Table 1 provides a summary (not exhaustive) of these efforts; Zimmerman recently provided a similar summary [40]. International comparisons for radioactivity measurements between NMIs usually involve the distribution of samples from a master solution to the participating laboratories for assay. Since the assays are of the same solution, the determined massic activities are directly comparable. This type of comparison was carried out amongst several European NMIs [22–25]. Inclusion of non-European NMIs was impossible due to the logistic challenges involved in shipping short-lived samples to distant laboratories. An alternative comparison method was ultimately proposed, wherein laboratories used primary activity standards to calibrate pressurized ionization chambers [25,35]. NIST participation in this comparison will be discussed in more detail herein (Sec. 3.5).

This paper summarizes all NIST radioactivity standardizations performed on ^18^F since 1982 (the first of record at any NMI). As we discuss, the compilation of this summary was motivated by a new standardization, reported in 2014 [41], with results that were discrepant with some past NIST standardizations by 4 %. Internal instance to instance consistency over a period of approximately 10 years had bolstered confidence in the NIST standard. The new standardization, however, is based upon a more extensive battery of robust primary methods than any previous efforts, and brings the NIST ^18^F standard into substantially improved accord with other NMIs. This review is intended as a companion to the 2014 report [41], and we hope that by collecting all available data and examining possible sources of bias, we can better understand the sources of the discrepancies. To date, we have eliminated many possible sources of bias, and we will discuss those that remain.

Precise standards for ^18^F activity are foundational for the advancement of quantitative molecular imaging. With this review, NIST is attempting to provide scientific and commercial stakeholders with as much information as possible regarding the reason for the change in this important standard.

The remainder of this paper is divided into four sections (2 through 5). Section 2 covers the various techniques used to standardize ^18^F, with special attention paid to the variables that could potentially introduce unexpected bias to the measurements. Section 3 covers the history of ^18^F standardizations at NIST. The specific experiments are placed in context, and a review of the techniques and their implementation is provided. In some cases, the benefit of hindsight allows for the identification of specific problems in the experiments. Section 4 attempts to summarize the perspective gained from this thorough review, including a discussion of the various sources of potential bias that we have identified. Section 5 offers some brief concluding remarks.

## 2. Techniques

In the general introduction to its chapter on “Fundamental or Direct Measurements of Activity in Radioactive Decay”, the NCRP Report No. 58 provides a summary of what makes a measurement fundamental [42]. In general, fundamental or primary methods are those that rely on nuclear decay data but not calibration data, with methods based on efficiency extrapolation “regarded as the most reliable” [42]. However, a “self-contained” calibration method like the efficiency-tracing method described in Sec. 2.1 can be considered primary because it is only weakly dependent on another radioactivity standard, underscoring that “the dividing line between direct and indirect methods can be tenuous” [42]. In general, we consider measurements made using the methods presented in Secs. 2.1–2.3 to be primary, while activities determined via the remaining methods are considered derived or secondary.

For short-lived radionuclides like ^18^F, the calibration factors developed for secondary methods provide a critical repository for the results of primary standardizations. Primary measurements made years or decades apart can be compared by appeal to secondary standards.

### 2.1 Liquid Scintillation Counting and CIEMAT/NIST Efficiency Tracing (CNET)

Liquid scintillation counting (LSC) is at the heart of many of the primary standardizations performed at NIST. Reference [43] provides a good review of the application of LSC techniques to radionuclide metrology. In general, LSC involves the conversion of ionizing radiation into visible light via interactions with an organic fluor suspended in a LS “cocktail”. The organic cocktail usually contains various surfactants (which micellize the aqueous radionuclidic sample), a fluor, and an additive that shifts the wavelength of the scintillation light towards the efficiency peak of the photomultiplier tube (PMT).

#### 2.1.1 CNET Technique

The CIEMAT/NIST efficiency tracing (CNET) method [44,45] of LSC relies on nuclide-specific efficiency calculations for a system of two PMTs in coincidence. This experimental configuration is typical of commercial LS counters. Efficiencies are calculated for the nuclide of interest and for a tracer nuclide, usually ^3^H, for which a standard is available. In practice, the counting efficiency for the nuclide of interest (*ε*_F-18_, in the present context) for a particular scintillation cocktail composition is linked to the counting efficiency of the tracer (*ε*_H-3_) through a measure of the quench indicating parameter (QIP). Two carefully matched (*vide infra*) series of LS cocktails containing the nuclide of interest and the tracer are prepared. Efficiency variation, typically achieved via chemical quenching with chloroform or nitromethane, across the series provides data on the variation of *ε*_H-3_ with QIP. A computer model is used to relate the measured *ε*_H-3_ for a given QIP to *ε*_F-18_ for the same QIP and the activity is then calculated by dividing the measured count rate by the model-derived *ε*_F-18_.

#### 2.1.2 CNET Variables

CNET results can be affected by the model used, the preparation and composition of the LS cocktails, and various aspects of the counting conditions including count rate and various instrumental parameters.

The relationship between the efficiency curves for the tracer and nuclide of interest is typically determined using one of several computational models [46–54]. Absorbed energy spectra are calculated from nuclear data input by the user using either analytical or stochastic approaches, depending on the specific computational program. For some nuclides, especially those that decay by electron capture (EC), model-dependence can be significant. F-18 has a small EC branch (3.14 %, [1]) that is usually considered to contribute negligibly to its LS counting efficiency (see Fig. 1 for the decay scheme). The efficiency of the β^+^ branch is very close to 100 %. Assuming 100 % β^+^ efficiency and neglecting the EC branch altogether gives a maximum discrepancy from MICELLE2-calculated *ε*_F-18_’s of only 0.36 % for *ε*_H-3_ > 0.2. (MICELLE2 is a computer code for calculating LSC efficiencies [51].) Agreement between models that include EC contributions and quenching is even better.

In the computational models currently in most widespread use, the treatment of quenching— specifically the computation of the ionization quenching function—is often the largest source of uncertainty. The ionization quenching parameter (Birk’s parameter, *kB*) is treated largely as an adjustable parameter, selected to provide the best fit of the model to the experimental data. If a model is based on a quenching function that fails to accurately represent the quenching characteristics of a sample (most common in the low energy portion of the function), the calculated efficiencies will be biased. Experiments have established a range of approximately 0.007 cm·MeV^−1^ to 0.015 cm·MeV^−1^ for LS cocktails [55]. For some nuclides, *kB* selection can have a significant effect on the recovered activity. The high counting efficiency for ^18^F results in negligible dependence on selected *kB*; MICELLE2 calculations reveal < 0.01 % difference in the ^18^F counting efficiencies calculated with *kB* = 0.011 and *kB* = 0.013.

Cocktail selection and preparation can be very important in CNET experiments. Cocktail stability is sensitive to the composition of the sample solution. A variety of scintillants are therefore commercially available, with unique combinations of solvent and surfactants designed to accommodate a wide variety of solution compositions and volumes. Poor scintillant selection or cocktail formulation can result in an unstable cocktail for which the counting efficiency decreases significantly with time [56]. The drop in efficiency can result from the radionuclide adsorbing on the walls of the LS vial. Or, in an inappropriate cocktail, highly ionic or strongly acidic or basic formulations can result in a chemical degradation of the cocktail. Such degradation might affect the fluorescence efficiency of the fluor or alter the solution’s quenching function. In practice, while the specific causes of cocktail instability are often unknown, its effects have been observed at numerous laboratories, spurring the development of many idiosyncratic recipes (varying in both cocktail content and in the preparation steps) for stable cocktails.

For ^18^F LSC cocktails, one can imagine a number of mechanisms for cocktail instability. In target water or fluoride samples, the highly reactive fluoride ions might interact strongly with the glass walls of LS vials, causing a reduction in efficiency. Use of a carrier solution with a sufficiently high concentration of F^−^ might help to overcome this problem, but would then increase the likelihood of deleterious chemical reactions in the cocktail (altering fluorescence efficiency and/or the quench curve). For [^18^F]FDG samples, chemical effects would be expected to be less severe, but solubility problems might still arise. Cocktail stability could also be affected by changes in ionic strength owing to dilution and diluent selection (water, buffer, saline, or halide salt solution).

The short half-life of ^18^F provides only a narrow window of time during which samples will give useable count-rates, making the detection of cocktail instability more difficult. If samples are prepared and then left to decay before counting, an early, precipitous drop in efficiency might escape notice. In addition, in CNET experiments with short-lived nuclides, the tracer series is often counted a day before or after the series containing the nuclide of interest; any cocktail instability effects would then be compounded with a potentially extreme departure from ideal cocktail matching (discussed presently).

Even if stability is achieved, cocktail formulation can still give rise to problems in CNET. The importance of precise cocktail matching was only fully appreciated after the technique had been in wide use for more than a decade [57–59]. Reliance on the QIP as a bridge between the counting efficiency of the tracer and the nuclide of interest means that the quenching mechanisms in the tracer and unknown cocktail series must be identical (and, as much as is practicable, arise from one variable). If, for example, the aqueous fraction is significantly different in the two series, it is possible to achieve identical values for the QIP on two different quench curves, resulting in erroneous estimates for the counting efficiencies [58,59]. This potential pitfall underscores both the need for careful cocktail matching (in order to mitigate these effects) and for extensive characterization of the efficiency curves via efficiency variation (in order to detect them). For high energy β^−^ and β^+^ emitters (including ^18^F), counting efficiencies are relatively high and thus relatively independent of cocktail quenching; for these nuclides, effects of cocktail mismatch are expected to be so small as to be practically undetectable.

Currently at NIST, emphasis on precise cocktail matching is such that the QIP link could be ignored since quenching in the matched sample and tracer series is effectively identical. The relationship between the sample and tracer series is thus compositional, relying on, for example, the mass of the added quenching agent, instead of the QIP.

In addition to model-dependences and cocktail-based variations in efficiency, CNET can be affected by certain aspects of the counting conditions. Instrumental nonlinearities can come into play in experiments with short-lived nuclides since counts are often taken with a very wide range of count rates. To compound the problem, counts on the long-lived tracer will not suffer from these effects. It is therefore important to be aware of any trends or discontinuities in the CNET-derived activities as a function of count rate. Other instrumental factors such as the low-energy threshold, deadtime, and energy binning can affect measured efficiencies. For this reason, when possible, it is preferable to acquire data on multiple LS counters [60].

### 2.2 Triple-to-Double Coincidence Ratio (TDCR) Liquid Scintillation Counting

#### 2.2.1 TDCR Technique

The use of the triple-to-double coincidence ratio (TDCR) LSC method for activity standardizations has been reviewed in depth by Broda [61]. In its simplest form, the double and triple coincidences in a system of 3 PMTs are measured, and the TDCR, *K*, is calculated from

$$K=\frac{N_{T}}{N_{D}}=\frac{\varepsilon_{T}}{\varepsilon_{D}}$$ (1)

where *N*_T_ and *N*_D_ are the count rates for triple and double coincidences of scintillation photons, respectively, and *ε*_T_ and *ε*_D_ are the counting efficiencies for triple and double coincidences, respectively. By varying the counting efficiency via gray filters, chemical quenching, or PMT defocusing, a curve can be built. As *K* approaches unity, the individual efficiencies also approach unity and *N*_T_ and *N*_D_ approach the disintegration rate. In modern applications, the “enhanced TDCR method” [62], which accounts for PMT inequality but requires a more sophisticated computational model, is often applied. The computer model predicts a relationship between *K* and *ε*_D_. Activity is calculated by dividing *N*_D_ by this derived *ε*_D_.

The NIST TDCR spectrometer has been described by Zimmerman *et al*. [63], with later modifications to the sample chamber described by Bergeron and Zimmerman [64]. Coincidence and extending deadtime logic are realized using either a MAC3 (for “module d’acquisition de coíncidences triples”) unit [65] or a field programmable gate array (FPGA; National Instruments model 7830R^1^)-based system. The FPGA-based system is of NIST design, utilizes LabVIEW code for the implementation of the counting logic, and can be run simultaneously with the MAC3 system.

#### 2.2.2 TDCR Variables

TDCR results can be affected by the model used, LS cocktail instabilities, and aspects of the counting conditions including count rate and various instrumental parameters. While many of the variables involved in TDCR overlap with those discussed for CNET (*vide supra*), there are additional factors and complications unique to this technique.

As more NMIs have adopted TDCR as a primary method, the number of computational approaches to its application has grown. While a few programs have been more widely used [51,66,67], it has been very common for different labs to develop their own codes to meet their specific needs. In a recent international comparison [68], a single set of data (measured at NIST with a ^99^Tc source) was distributed to nine laboratories for analysis. No two labs used the same version of the same code, but all labs that properly input the nuclear data to their model achieved reasonable results. The most serious difficulties surrounded entry of the beta spectrum shape factor. While model-dependences could be more severe in some cases (such as low-energy EC nuclides, where a detailed description of atomic rearrangement processes is crucial), they were insignificant for ^99^Tc, which decays via β^−^ emission (*E*_max_ = 293.8 keV) [69]. The high counting efficiency for the β^+^ branch in ^18^F decay (and the fact that the contribution of the EC branch to the counting efficiency is negligible) makes model-dependence insignificant.

In general, the models (and their implementation in computer codes) underlying CNET and TDCR are the same. Most codes use Birk’s function to describe quenching, with *kB* being generally used as an adjustable parameter. TDCR ^18^F counting efficiencies are not significantly influenced by *kB* selection.

As in CNET, cocktail stability poses problems in TDCR. If the radionuclide of interest adsorbs onto the walls of the LS vial or precipitates out of the solution, the “misplaced” material will have a substantially reduced counting efficiency compared to that calculated by the model for all decays, and the measured activity will appear low. TDCR should be less sensitive than CNET to chemically-induced reductions in cocktail fluorescence efficiency, but changes to the quench function could still bias results. Because TDCR does not rely on comparison with a tracer or QIP-based efficiency calculations, it does not suffer from the effects involving initial or evolving cocktail mismatch (Sec. 2.1). As a sort of tradeoff, since no QIP or tracer efficiency is monitored, there are less data upon which to base an evaluation of cocktail stability. With a short-lived nuclide like ^18^F, a TDCR experiment might be less likely to detect a time-dependent drop in efficiency than a CNET experiment would be. This is compounded by the fact that the NIST TDCR system does not have an automatic sample changer that would facilitate repeated measurements on sources over a long period of time (as is common practice on the commercial counters used for CNET). It is generally assumed that the TDCR method, by providing a direct determination of the efficiency with each measurement, should be immune to cocktail instability effects. However, certain mechanisms of instability, especially those that result in an effective change of the counting geometry (e.g., the radionuclide is settling out of the solution), will not be accounted in the TDCR model and may thus prove problematic.

Like CNET, TDCR is sensitive to certain aspects of the counting conditions. The challenges of keeping count rates in the counter’s linearity range with a short-lived nuclide are the same for any counting method. Other instrumental factors such as the low-energy threshold, deadtime, and coincidence resolving time (CRT) can affect measured efficiencies [64,70–73]. These effects would all be expected to be small for the ^18^F case. Where possible, however, their characterization and minimization are certainly advisable.

### 2.3 Live-Timed Anticoincidence Counting (LTAC)

#### 2.3.1 LTAC Technique

The live-timed anticoincidence counting (LTAC) technique provides robust primary measurements for counting β–γ decays. The method uses live timing and extending dead-time circuitry to eliminate the need for dead-time and resolving-time corrections [42,74]. The experimental configuration allows for counting γ events not correlated with β events, so that the LS inefficiency is approximated by *Y*, the ratio of anticoincident γ events to all γ events. As in TDCR, the LTAC method provides a direct determination of the (in)efficiency with each measurement. From the basic anticoincidence equation for pure β–γ decay

$$N_{\beta }=N_{0}+g(Y)$$ (2)

where *N_β_* and *N*_0_ are the beta emission and decay rates, respectively, and *g* is a polynomial function, where *N_β_* ➔ *N*_0_ as *Y* ➔ 0. Measurements with varying lower-level discriminator settings (effectively varying the beta detection efficiency) provide a range of *Y*, so that *N_0_* can be determined via simple extrapolation. The specific implementation of LTAC at NIST involves a hemispherical LS source placed on a PMT that is raised into a NaI well-type detector and has been described in [75,76].

LTAC is well-suited to the standardization of ^18^F, with an extrapolation equation

$$N_{\beta^{+}}=N_{0}P_{\beta^{+}}(1-kY)$$ (3)

enabling the straightforward calculation of activity from the extrapolation intercept and the positron branching ratio, $P_{\beta^{+}}$ In the ^18^F experiments, the correction for the γ-detection efficiency of the LS detector, *k*, is constant over the extrapolation, as evident in a linear extrapolation [41].

#### 2.3.2 Variables

The simplicity of the LTAC model, which does not rely upon any explicit calculation of scintillation or counting efficiency, makes the technique extremely robust. For LS-based LTAC, results can be affected by aspects of LS cocktail preparation and composition. Counting conditions can also come into play.

Because LTAC does not require a microscopic model of scintillation and quenching, cocktail instability is not as great a concern. The precise mechanism by which inefficiency in the β channel is achieved and varied is, in principle, irrelevant to the LTAC model for a single-β^+^-branch decay such as ^18^F. However, if both the LS and NaI efficiency are a function of position within the vial (e.g., if the ^18^F is settling out of solution), then the extrapolation would not necessarily be linear.

LTAC is, of course, sensitive to the presence of impurities in a sample. An accounting of impurities in both the LS and NaI count rates is thus essential to obtaining accurate results. Similarly, precise background subtraction is very important. Since the NIST LTAC instrument relies on a single PMT for the detection of scintillation light for the β channel, the LS background and potential for afterpulsing are greater than is typical of most commercial LS counters which feature two PMTs counting in coincidence mode. To reduce the afterpulsing to an insignificant level, an extending deadtime of 50 µs is applied. To reduce sample-to-sample background variability, sources and blanks are of identical volume and are handled so that both are exposed to identical light conditions during source preparation. To reduce UV-induced luminescence, sources are prepared under incandescent light instead of the usual fluorescent light, and all sources are allowed to dark-adapt for several hours after preparation. Finally, for ^18^F, the lower-level discriminator was limited to about 10 keV, where the LS background was only about 3.5 cps. Using these precautions, the uncertainty due to impurity and background were less than 0.1 % [41].

In most LTAC experiments, the largest source of uncertainty is due to the extrapolation. This uncertainty can be reduced somewhat by carefully monitoring the residuals of the fit for any trends that might indicate an inappropriate selection for the polynomial, *g*. Often, good residuals can be achieved for more than one fit, e.g., linear and quadratic. In such cases, it has been common practice at NIST to take the mean of the two results and incorporate the standard deviation into the uncertainty. For long extrapolations, the uncertainty due to the fit can be significant, so it is always preferable to acquire experimental points that cover a large range of inefficiencies and minimize the magnitude of the extrapolation.

The effect of the γ-ray sensitivity of the LS counter, represented by *k* in Eq. (3), depends on the choice of the γ-gate or gates used in the extrapolation. The extrapolation is linearized by judicious choices of the gates [77,78]. It has been common practice at NIST to use the ideally-linearized gate for the activity value and to incorporate the standard deviation of the results from using other gates, which have apparently linear extrapolations, into the uncertainty [41,79].

### 2.4 Gamma Spectrometry

For most primary standardizations at NIST, γ-emitting impurities are inventoried and assayed via gamma spectrometry. In recent years, these measurements have been performed with a battery of HPGe detectors with well-characterized efficiency curves [80]. For γ-emitting impurities, detection limits are typically < 10 γ·s^−1^ from 15 keV to 2000 keV. For ^18^F, an activity was determined with an uncertainty of 1.3 %.

NIST also operates a 4πγ (NaI) integral counting system consisting of two 20 cm (“8-inch”) well detectors sandwiched together. The efficiency curve for this system is based on a Monte Carlo simulation that was constrained to the measured dimensions of the apparatus and to primary standards of ^57^Co, ^60^Co, and ^99m^Tc. The efficacy of such an efficiency curve determination has been demonstrated for a HPGe system [81]. The efficiency for ^18^F is not expected to fall on the calibration curve, since the β^+^ decays in flight and the annihilation γ-rays are angularly correlated. These effects are accounted for using Monte Carlo simulation and by experiments of the sensitivity of the instrument to the position of the source.

### 2.5 Ionization Chambers

Calibrated ionization chambers (ICs) are commonly used as repositories for derived or secondary standards of short-lived radionuclides [42,82]. In clinical settings, similar instruments provide the basis for activity measurements, usually relying upon manufacturer-provided calibration factors or “dial settings” to convert measured currents to activities to be displayed directly in units of Bq or Ci.

#### 2.5.1 NIST Chamber “A”

The NIST pressurized ionization chamber “A” has been used since the 1960s to maintain activity standards via calibration factors (K-values, *vide infra*) [83]. Details of the chamber and its operation have been discussed in a recent publication that addressed the ramifications of the discovery of a slow drift in the height of sample placement resulting from a slipping positioning ring [84].

Briefly, chamber response is measured relative to a ^226^Ra reference source (RRS), to give a ratio *R*, which is related to the activity (*A*) by the calibration factor (K, which we refer to as the K-value) according to

K=A/R (4)

In contrast to a calibration factor based solely on the relationship between chamber response and activity, the ratio-based K-value is insensitive to variations in the electrometer response over time. Thus, the measurements are less sensitive to environmental factors such as humidity and temperature, and the K-values are not electrometer-dependent.

The first K-value for ^18^F was calculated in 1982 based on the chamber’s known response to the positron emitter ^22^Na in units of positrons per second (rather than Bq). The methodology behind calculating theoretical IC calibration factors is well-established, and was reviewed by Schrader in [82].

Recently, another IC, the NIST automated ionization chamber or “AutoIC” has been added as a repository for secondary standards [85].

#### 2.5.2 Vinten 671/Keithley 6517

NIST also maintains a Vinten 671 IC (VIC), commonly referred to as an “NPL-type” chamber which has a sister IC at the National Physical Laboratory (UK) [86]. The NIST VIC is read by a Keithley 6517 electrometer which interfaces with a PC running a LabVIEW program for data acquisition.

Calibration factors (K_VIC_) are stored in units of pA·MBq^−1^, without reference to a RRS. However, contemporaneous QC measurements of a RRS assure that no unexpected drift in the electrometer response has occurred.

The relationship between the chamber at the NPL and the NIST VIC allows for comparison of calibration factors (though often volume corrections are necessary since NPL typically measures 3 mL in a 5 mL British standard ampoule, while NIST measures 5 mL in a 5 mL NIST standard ampoule; the ampoule type has minimal effect on the calibration factors). This link allows indirect comparisons of activity standards.

#### 2.5.3 Commercial “Dose Calibrators”

Commercially available reentrant ICs are commonly referred to as “dose calibrators”, a potentially confusing appellation (the instruments calibrate activity, not dose) that has frustrated some scientists for years [42]. “Radionuclide calibrator” (commonly used in the U.K.) and “activity calibrator” have been proposed as more accurate alternatives, but the medical association with dosage has helped to enshrine “dose calibrator” in common U.S. usage. With the incorrectness of the term duly acknowledged, we adopt the common usage herein and refer to commercially available reentrant ICs as “dose calibrators”.

When the appropriate calibration factor or “dial setting” (DS) is used, a dose calibrator reports an accurate value directly in terms of activity. NIST has reported on dose calibrator DS determinations for numerous medically important short-lived nuclides, including ^18^F [38,39,87,88]. The publication of calibration factors is one means of providing measurement guidance for short-lived nuclides. NIST has also developed methods for disseminating standards for short-lived nuclides via long-lived surrogates [87]. In the case of ^18^F, NIST has taken both of these approaches.

NIST’s collection of dose calibrators has grown over time. While some chambers have been retired due to obsolescence or gradual failure, others have proven to be robust repositories for secondary standards. Regular quality control tests including (at least weekly) constancy checks with a RRS and (approximately annual) linearity checks with short-lived nuclides assure that chamber response remains constant over time, thus assuring that previously determined DS’s remain valid.

## 3. Results of Primary Standardizations of ^18^F at NIST (1982–2013)

**Fig. 2:**
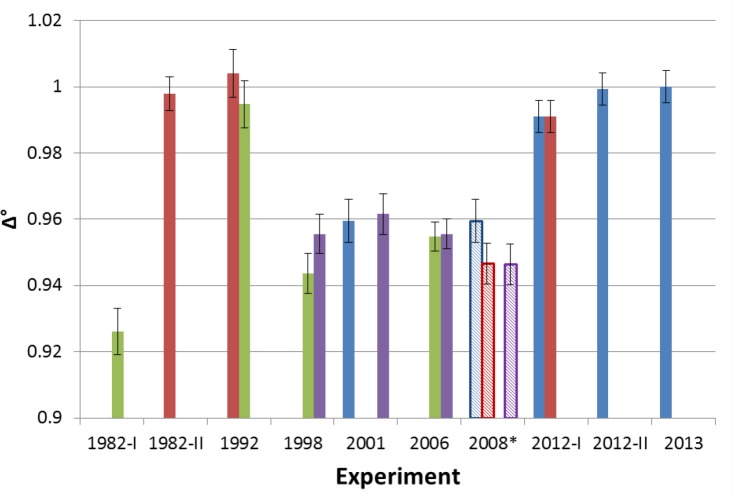
A comparison of the results of NIST LSC-based standardizations of ^18^F, normalized to the 2012 LTAC-based standard. See text (Sec. 4.1 and Eq. (5)) for the derivation of Δ°. The bar colors identify the specific ionization chambers used to establish links between the experiments. Blue – VIC; Red – Chamber “A”; Green – Capintec dose calibrator, DS = 439; Purple – Capintec dose calibrator, DS = 472 to 477. The 1982-I link was calculated from the average of the NIH CRC-16 and CRC-30 measurements, with the uncertainty bars corresponding to the standard deviation on those measurements. The 2006 Capintec links are based on measurements with a CRC-15R dose calibrator. All other Capintec links are based on measurements with the NIST CRC-12. Uncertainty bars are estimated standard uncertainties (*k* = 1); unless more detailed uncertainty information was available, the uncertainties are simply taken from the standard uncertainty on the corresponding primary activity standard. Chamber “A”-based links do not include chamber-height corrections. *The 2008 links are given in outline to emphasize that no LSC measurements were performed in 2008; the 2008 activity was based on the VIC measurements, using the K_VIC_ determined from the 2001 LSC measurements. Thus, the blue VIC bars from 2001 and 2008 are identical.

At various times over the years, NIST has applied all of the methods in Sec. 2 to the determination of ^18^F activity. In this section, we attempt to compile relevant data on the ten primary standardizations performed at NIST between 1982 and 2013; the documentation of methodology is generally strong and the use of secondary techniques permits a reliable comparison of results. The experimental methodologies applied in the studies varies substantially, owing to different goals and motivations, changes in personnel, improvements or changes in available techniques, and, in some cases, different locales. Table 2 provides a summary of the methods applied in all of the experiments and illustrates the specific linkages between experiments afforded by ionization chamber (IC) measurements. Table 3 gives specific details on the liquid scintillation measurements made in each experiment, allowing for an easier comparison of the variables outlined in Sec. 2.1.2. Figure 2 provides a comparison of the normalized results of the standardizations using secondary linkages as described in Sec. 4. Unless stated otherwise, all uncertainties are reported as combined standard uncertainties with *k* = 1.

### 3.1 1982-I

In May of 1982, NIST collaborated with the National Institutes of Health (NIH) on a primary standardization of ^18^F. The goal was to establish a primary standard and determine a K-value for NIST chamber “A” so that future calibrations could be linked to the primary standard. The results of this standardization were not published, and it can be considered a preliminary study leading to the standardization described in Sec. 3.2. LS counting efficiencies were calculated assuming a 100 % counting efficiency for positrons, combined with a β^+^ branching ratio of 0.969 and assuming negligible counting efficiency for the EC branch. No record of the scintillant could be found, but the diluent was 0.1 mol·L^−1^ KI. LS sources were prepared from a diluted solution and counted shortly after preparation. Six LS vials were prepared; 3 at 10 mL and 3 at 18 mL.

No efficiency tracing technique was applied; indeed, in 1982, the concepts behind CNET were only just being conceived. Integral count rates were measured as a function of the lower level discriminator setting (8 points for each of 6 sources), and an extrapolation (second order polynomial) to zero provided the count rate for activity calculations.

The ^18^F source in this experiment was [^18^F]KF, produced at NIH. Point sources were prepared and measured on a Ge(Li) detector, and the β^+^ rate was determined; no explicit record of an impurity determination could be found, but no ^48^V or any other impurity common to ^18^F solutions was noted. The LSC-based activity determination did not include impurity corrections. Since the length of the time period between the end of bombardment (EOB) and the LSC measurements is unknown, it is impossible to estimate any measurement bias resulting from unaccounted impurities (Secs. 3.4 and 4.3.3 provide more details on impurity corrections).

A NIST standard 5 mL ampoule containing nominally 5 g of the [^18^F]KF in KI buffer solution was measured in chamber “A”. At the time, the data were analyzed using a K-value derived for 511 keV photons (K_511_), giving an activity ≈ 25 % lower than the LSC-based value. Later in 1982, a theoretical K-value was calculated for ^18^F (Sec. 3.2), but the chamber “A” data originally analyzed with K_511_ were never reanalyzed.

No dose calibrator measurements were made at NIST for this experiment, but measurements on a 5 mL vial containing approximately 2.8 g of the [^18^F]KF in KI buffer solution were performed at NIH. Measurements on a Capintec CRC-30, a Capintec CRC-16, and an unknown Capintec model (all using DS = 439) returned activities that were an average of 9.1(1.3) % higher than the LSC-based value.

### 3.2 1982-II

In July of 1982, a second experiment was performed in collaboration with NIH [37]. The results of the primary standardization were applied to the calibration of a dose calibrator, a PET scanner, and a NaI(Tl) well-type gamma counter at NIH. LS counting efficiencies were calculated as described in [37] to yield *ε*_F-18_ = 0.951 to 0.957. Two scintillants, Instagel and Picofluor 15 (Packard Instrument Co., Downers Grove, IL), were used with several scintillation vial types and levels of filling. Efficiency variation was thus achieved not by chemical quenching, but by variation of meniscus height, total aqueous fraction, and vial size/material. LS sources were prepared from a diluted solution (the diluent was distilled water) and counted shortly after preparation.

The discriminator extrapolation method mentioned in Sec. 3.1 was applied to a series of ^18^F and ^22^Na LS sources. Augustín Grau Malonda (CIEMAT, Madrid) participated in the preparation of the sources and the LS counting. The initial concepts leading to the development of CNET were discussed during the time of these measurements, but the published results for ^18^F were based on the discriminator extrapolation and the assumption of 100 % counting efficiency for positrons. In parallel, Ge(Li) measurements were performed on solid point sources.

According to Ref. [37], the ^18^F source used in these experiments was “carrier-free fluoride ion in normal saline (0.9 % NaCl w/v)”; however, the shipping invoice from July 1982 indicates that the material was [^18^F]FDG. The ^18^F was produced at the Naval Research Laboratory, which makes the delivery of [^18^F]FDG unlikely. However, the material was delivered to NIH prior to being transferred to NIST, so it is possible that [^18^F]FDG was prepared from the fluoride solution at NIH. Whether the sample was [^18^F]FDG or fluoride solution, no gamma-emitting impurities were observed in the Ge(Li) measurements, and no impurity corrections were made. Since the length of the time period between the EOB and the LSC measurements is unknown, it is impossible to estimate any measurement bias resulting from unaccounted impurities (more details in Secs. 3.4 and 4.3.3).

In addition to LSC, measurements were made by Ge(Li) and 4πγ IC (NIST chamber “A”). The chamber “A” results were obtained using a K-value based on the chamber’s response curve and measurements with a ^22^Na standard. The Ge(Li) and chamber “A” assays agreed with each other to ≈ 0.5 %; the LSC-derived activity was 0.3(9) % to 0.8(8) % lower. No records of dose calibrator measurements in July 1982 could be found.

### 3.3 1992

In 1992, NIST collaborated with the PET center at NIH to provide calibrations of dose calibrators and a NaI(Tl) well-type “gamma counter” at NIH. LS counting efficiencies were calculated assuming a 99.96 % counting efficiency for positrons, combined with a β^+^ branching ratio of 0.969 and assuming negligible counting efficiency for the EC branch. No record of the scintillant could be found, but the diluent was a phosphate buffer solution. LS sources were prepared from a diluted solution and counted shortly after preparation, approximately 8 hours after EOB.

Records do not indicate the use of an efficiency tracing technique, but 5 of the 8 LS sources contained an additional 1 to 5 drops of phosphate buffer solution, resulting in a very small difference in the QIP across the series. This small efficiency variation had no systematic effect on the measured count rates; the standard deviation on count rates (normalized by mass) of the 8 samples in each cycle was 0.2 % to 0.4 %, whereas the average cycle-to-cycle variation for a single sample was 0.6 %.

The ^18^F source in this experiment was [^18^F]FDG, purified at NIH. Point sources were prepared for impurity measurements via Ge(Li), but no record of the results exists. For the LSC activity determinations, it does not appear that any impurity corrections were made. Based on the 1998 results (Sec. 3.4) and the relatively short period between the EOB and measurement time, we can conservatively estimate that the bias in the calculated activity due to unaccounted impurities should be less than + 2 %.

The ^18^F solution was also measured on the NIST chamber “A”, using the ^22^Na-based theoretical K-value determined in 1982 (Sec. 3.2). The massic activity value derived from LSC was 0.87(65) % lower than the value derived from chamber “A” measurements. Measurements on two NIST standard 5 mL ampoules on the NIST Capintec CRC-12 at DS = 439 gave readings that were 1.81(3) % to 2.43(3) % lower than the LSC-derived activities. Dose calibrator measurements were also made at NIH, but no record of the results could be found.

As illustrated in Fig. 2, according to the chamber “A” link, the LSC-derived activity was 0.6(9) % higher than in 1982-II; according to the Capintec dose calibrators (not the same chamber or geometry in both experiments), it was 7(1) % higher than in 1982-I.

### 3.4 1998

In 1998, NIST collaborated with the PET center at NIH on a series of experiments to determine dose calibrator dial settings for ^18^F in clinically relevant geometries [38]. LS efficiencies were calculated with EFFY [46], and the scintillant was Ultima Gold AB (PerkinElmer, Waltham, Massachusetts; UGAB). The diluent was saline, and cocktails were prepared with high activity solution and allowed to decay for approximately 12 h prior to counting.

A CNET code (EFFY4; [46]) was used to calculate efficiencies, but no efficiency variation technique (i.e., chemical quenching) was applied. At low aqueous fractions (0.1 %), poor measurement repeatability and reproducibility (1.0 % and 0.30 %, respectively) was attributed to cocktail instability. With “normal” aqueous fractions of nominally 9 %, repeatability and reproducibility improved to 0.56 % and 0.19 %, respectively. For the low aqueous fraction series, *ε*_H-3_ = 0.36 to 0.37, while for the “normal” aqueous fraction series, *ε*_H-3_ = 0.40 to 0.41. The observation in Ref. [38] that UGAB cocktails with aqueous fractions less than about 5 % are unstable is supported by recent dynamic light scattering measurements on this commercial scintillant that indicate a stark change in micellar behavior near this threshold [89].

The 12 h delay between cocktail preparation and counting would have made it difficult to spot any trends in counting efficiency with time. Even if such effects had persisted past the first 12 h, they would have been completely obscured by impurity effects.

The ^18^F source in this experiment was [^18^F]FDG, purified at NIH. Zimmerman *et al*. discuss a ^48^V impurity, identified by HPGe, in some detail in Ref. [38]. A 640(26) % discrepancy between the impurity activity determined by HPGe and that derived from LS data (assuming *ε*_V-48_ ≈ 0.60, as calculated by EFFY4) was noted, and the inadequacy of the final impurity correction was noted and demonstrated in a plot (Fig. 2 of Ref. [38]) showing an exponential increase in recovered massic activity (*C*_A_) at low count rates. As the ^18^F decays, at low count rates, the ^48^V impurity (*t*_1/2_ = 15.9735(25) d [90]) becomes increasingly significant. The large uncertainty on the ^48^V activity made an exact accounting of the impurity extremely difficult, resulting in an overestimation of the ^18^F *C*_A_ that increased with time. Only the first few counting cycles were used for the determination of the standard value for *C*_A_, and thus an estimated standard uncertainty of 62 % in the determination of ^48^V activity resulted in only a 0.08 % standard uncertainty on the calculated *C*_A_.

**Fig. 3:**
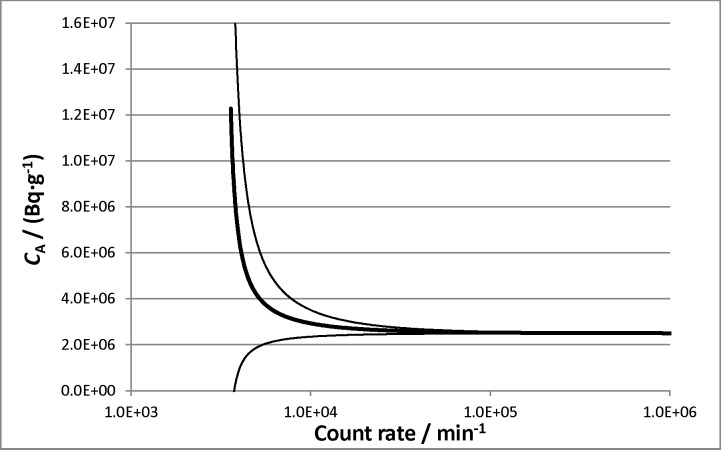
Calculated by attributing the difference in the ^48^V impurity determinations by liquid scintillation counting and HPGe to ^3^H (see text, Sec. 3.4, for details), this plot qualitatively reproduces the observed rate-dependence of the massic activity from Fig. 2 of Ref. [38]. The calculations assume efficiency values consistent with the 1998 experiments: *ε*_F-18_ = 0.9686, *ε*_V-48_ = 0.6, and *ε*_H-3_ = 0.41. The bands surrounding the dark line represent the bounds calculated by propagating the 1998 estimated uncertainty on the activity of the ^48^V impurity. Since only the first few cycles were used for the standard activity determination, the average and maximum biases stemming from the impurity misattribution are 0.25 % and 0.49 %, respectively.

Recent analyses have identified ^3^H as a very common radionuclidic impurity in ^18^F production [91–94]. Target water samples have been shown to contain approximately 100 times more ^3^H than all other radionuclidic impurities combined. Reinterpreting the 1998 results in light of this work—which is consistent with more recent measurements at NIST, see Sec. 3.10—explains the large discrepancy between the HPGe (which cannot detect ^3^H) results and the LS results. Typical LS efficiencies for ^3^H range from 0.4 to 0.5, and the long half-life (12.312(25) a [95]) would introduce an effectively constant background. At longer decay times, the underestimation of the half-life of the impurity would have resulted in an underestimation of the required impurity correction, leading to an overestimation of the impurity-corrected count rate, and thus an overestimation of *C*_A_. This qualitatively accounts for the results in Fig. 2 of Ref. [38] (see Fig. 3).

Because the activities reported in [38] are calculated only from the earliest counting cycles, the impact of the suspected ^3^H impurity is still expected to be fairly small. Assuming that the difference between the HPGe and LS values for the ^48^V impurity can be completely attributed to ^3^H, it is possible to calculate the bias introduced to the 1998 activity determination by the incorrect impurity analysis. For the counting period used in the activity determination, the average bias for the “normal” aqueous fraction series is 0.25 %. The bias increases with time, reaching a maximum of 0.49 % at the end of the counting period. For the low aqueous fraction series, the lower value for *ε*_H-3_ would result in smaller biases.

The calculated bias due to the possible incorrect attribution of ^3^H impurities to ^48^V would not change the calculated activity by more than its reported uncertainty. The total combined uncertainty on the massic activity determined in 1998 was 0.60 %, with the largest individual component (LS measurement repeatability, defined as the average standard deviation on the repeated determination of *C*_A_ for a single LS sample) contributing 0.56 %. As discussed in Ref. [38], uncertainties due to the uncertainty on the impurity correction are partially embodied in the uncertainty due to LS measurement repeatability.

The calculated standard activity measured via CNET in 1998 was applied to IC calibrations at NIST and at NIH for several geometries. While syringe and dose vial geometries are very important to clinicians, in the context of this review, the settings for the NIST standard 5 mL ampoule geometry are the most important. NIST determined DS values of 477(7) and 474(6) for each of two ampoules on the CRC-12 instrument, and 472(7) and 470(7) for the ampoules on the CRC-35R instrument. The uncertainties on the dial settings were obtained by propagating the expanded (*k* = 2) uncertainty in units of activity through the fitting equations used to determine the dial settings.

The difference in the activity measured using the NIST-determined dial settings and that measured using the manufacturer’s recommended “439” dial setting ranged from 6.0 % to 7.3 %. Measurements at NIH using the manufacturer’s “439” dial setting for two Capintec dose calibrators gave results that were 7.8 % to 8.3 % higher than the NIST standard activity. The report describing these findings concluded, “This clearly demonstrates the need to experimentally determine calibration factors for each radionuclide for each geometry in each individual dose calibrator in order to achieve the most accurate results [38].”

As illustrated in Fig. 2, according to the Capintec dose calibrators, the LSC-derived activity was ≈ 2.5 % higher than in 1982-I and ≈ 5 % lower than in 1992.

### 3.5 2001

In 2001, NIST participated in the CCRI(II)-K3.F-18 intercomparison [25,35]. The comparison methodology centered on the reporting of a VIC current for a ^18^F activity, to be converted to a calibration factor (K_VIC_) and scaled by a normalization factor calculated from contemporary VIC measurements on a ^68^Ge source [35]. The conversions, normalizations, and impurity corrections were handled by the organizing laboratory (NPL).

At NIST, CNET was performed using EFFY+EMI [46,47], and CN2000 [52] to calculate ^18^F counting efficiencies (*ε*_F-18_). The programs gave the same results: for *ε*_H-3_ = 0.4, *ε*_F-18_ = 0.96. LS measurements were made for two different dilutions of the master solution and the higher activity sources were measured ≈ 0.5 d after the lower activity sources. The average recovered activity for the high activity sources was 0.6 % higher than for the low activity sources. The average of the activities determined for the high and low activity sources was used for the comparison report.

Measurements were also made on several ICs. Using the DS obtained in the 1998 experiment, DS = 477, the CRC-12 gave an activity 0.6 % higher than the LS assay; using the CRC-12 current readout, a calibration factor of 8.647 pA·MBq^−1^ was determined. A K_VIC_ of 10.80(7) pA·MBq^−1^ was determined; this factor is 4.4(1.2) % higher than the volume-corrected NPL K_VIC_, 10.34(10) pA·MBq^−1^ [96]. Note that both the CRC-12 and VIC calibration factors were determined with impurity-corrected activities, but uncorrected chamber currents.

The ^18^F source in this experiment was target water, and ^48^V (0.17 % at the reference time) was detected as an impurity. At the time, no corrections were made for ^3^H impurities; it is possible that the presence— and partial misattribution—of ^3^H can account for some of the 0.6 % difference observed between the two dilutions. For the international intercomparison, impurity corrections were made by the organizing laboratory (NPL), and the precise nature of the data manipulations was not shared with the participants. The actual values reported for the intercomparison were the ^18^F activity, the VIC response in pA, the ^48^V impurity ratio, and the VIC response for a calibrated ^68^Ge source provided by the host laboratory. The ^18^F VIC response was scaled by a normalization factor calculated from the ^68^Ge VIC response by the host laboratory [35].

The discrepancy between the NIST-determined K_VIC_ and the NPL-determined K_VIC_ is of comparable magnitude to the discrepancy between the NIST value and the Key Comparison Reference Value (KCRV) for ^18^F. This is in keeping with the fact that the NPL was in excellent accord with the KCRV.

While certainly worrisome, at the time the ≈ 4 % discrepancy between the NIST standard and the KCRV was attributed to possible weaknesses in the comparison methodology (using different, imperfectly matched, ICs as a basis for the comparison; comparing NaF and FDG sources and making unclear impurity corrections). As we hope to make clear in this review, confidence in the NIST standard was fueled not by hubris, but by apparent internal consistency. In this particular instance, consistency with past standardizations was established through the NIST CRC-12 dose calibrator, which gave an activity reading that agreed with the CNET-based result (to within 0.6 %) using the “477” DS established in 1998 (Sec. 3.4).

### 3.6 2006

In 2006, NIST performed a standardization of ^18^F as part of a pilot program to establish regional secondary standards laboratories in the United States [39]. The work was performed at the “NIST/ORNL Intercomparison Metrology Laboratory” in the Quality division of the Oak Ridge National Laboratory (ORNL) in Oak Ridge, TN. This short-lived facility was intended to facilitate regional comparisons of ^18^F measurements, but suffered from a lack of interest/participation from stakeholders and was ultimately made obsolete by the development of long-lived surrogate standards for ^18^F calibrations [87].

As in 1998 and 2001, CNET provided the basis for the primary standardization performed at ORNL in 2006. LS efficiencies were calculated with CN2004. Ready Safe (Beckman Coulter, Fullerton, California) and Opti-Fluor (PerkinElmer) were the scintillants. The diluent was distilled water, aqueous fractions ranged from 0.5 % to 3.5 %, and efficiency variation was achieved via addition of nitromethane. Sources were counted approximately 185 minutes after preparation. The tritium tracer sources were prepared with inactive FDG (natural isotope form, not decayed [^18^F]FDG). Both cocktail formulations appeared to be stable with time.

HPGe measurements found no γ-emitting impurities. After 36 h of decay the ^18^F LS cocktails gave count rates that were indistinguishable from the background, indicating no significant β-emitting impurities (including ^3^H). The [^18^F]FDG used in these experiments was supplied from a radiopharmacy (PETNET Solutions, Knoxville, TN), and was clearly of much higher radionuclidic purity than the [^18^F]FDG used in the 1998 experiments.

The total combined uncertainty on the massic activity determined in 2006 was 0.43 %, with the largest individual component (LS measurement repeatability) contributing 0.33 %. Consistency with past standardizations was established through a dial setting determination on a Capintec dose calibrator at ORNL. As in 1998, determinations were made for a clinically relevant geometry, but the most important data for the purposes of this review are for the NIST standard 5 mL ampoule. A DS of 472(5) (*k* = 2) was determined for a CRC-15R dose calibrator; the difference between the activity reading at this NIST-determined setting and at the manufacturer’s recommended DS of “439” was 6.4 %. Geography precluded direct measurement of the ampoule in this experiment on a previously NIST-calibrated ionization chamber, and so the link between experiments was based on Capintec’s claim of < 2 % chamber-to-chamber variability. In the absence of a more direct link for comparison, then, the DS determination agreement (with the 1998 determination for the NIST CRC-12, to within the stated uncertainties) gave confidence that consistency between the NIST/ORNL result and previous NIST results had been achieved.

After the NIST/ORNL Intercomparison Metrology Laboratory project ended, the CRC-15R chamber used in the 2006 experiments was transferred to NIST, allowing for a direct comparison with the NIST CRC-12. Using a 2.5 MBq ^68^Ge source, we have established that these two chambers give activity readings that agree to 0.4 % at DS = 472, bolstering the validity of the previously [39] proposed link. Note that these measurements were made with the ^68^Ge source in the bottom of the dipper, as would always be the case for an ampoule. Also note that this CRC-15R (SN 157629) dose calibrator is not the same CRC-15R (SN 155544) used in most NIST dial setting determinations; however, the two CRC-15R calibrators agree in both geometries to < 0.4 %, comparable to the observed variation (0.2 % to 0.4 %) on the reading itself.

All available (then and now) evidence suggests consistency across the 1998, 2001, and 2006 standardizations.

### 3.7 2008

In 2008, NIST performed several ionization chamber measurements as part of the project to develop a calibration surrogate for an ^18^F-containing syringe [87]. No primary standardization was performed at this time. Instead, a NIST standard 5 mL ampoule was measured on chamber “A”, on the VIC, and on the Capintec CRC-12.

The activity determined from measurements on chamber “A” using the K-value derived in 1982 (Sec. 3.2) was 5.1 % higher than the activity determined from measurements on the VIC using K_VIC_ = 10.80(7) pA·MBq^−1^, as determined in 2001 (Sec. 3.5). A reassessment of this result in light of chamber “A” height effects [84] brings the chamber “A” result to within +3.96 % of the VIC result. The activity determined with the Capintec CRC-12 using DS = 475 was 0.92 % lower than the VIC result and 3.76 % lower than the chamber “A” result.

Again, no LSC measurements were performed in 2008. However, the ^18^F calibration factors used for the VIC and the CRC-12 were based on earlier (2001) NIST LSC results. In Fig. 2, 2008 links are established using the VIC-determined activity, so that the 2008 data are essentially adding links for the 2001 LSC determination. The 2008 experiment was the first time since 1992 that a chamber “A”-determined ^18^F activity was compared, albeit indirectly, with an LS-based value, revealing that the LSC-derived activity was 5.1 % lower than in 1982-II and 5.7 % lower than in 1992. It was also the first time that results from chamber “A” were compared directly with results from the VIC.

### 3.8 2012-I

In 2012, NIST undertook a new primary standardization of ^18^F [41] as part of an effort to link the calibration of the NIST PET-CT scanner directly to primary radioactivity standards. For the first time at NIST, CNET, TDCR, and LTAC were all performed on a ^18^F sample. Further measurements on the 8-inch NaI(Tl) and HPGe systems were also made. All work was carried out at NIST, and measurements were performed on a total of 8 ICs, including chamber “A”, the AutoIC, and the VIC. Measurement results are compared relative to the LTAC result, which was adopted as the basis for the new ^18^F activity standard [41].

The source was target water from Cardinal Health and HPGe measurements identified ^52^Mn, ^54^Mn, ^55^Co, ^56^Co, ^57^Co, ^96^Tc, ^183^Re, and ^195m^Hg as γ-emitting impurities. The presence of ^3^H was also suspected. All activity determinations included impurity corrections, but these were small (approximately 0.005 % at the mid-time of LTAC counting).

LS efficiencies for CNET and TDCR were calculated with MICELLE2 [51]. Comparison of the CNET efficiencies calculated with CN2004 [52] showed no difference to within the computed precision. Two scintillants, Ultima Gold AB (UGAB) and Hionic Fluor (PerkinElmer; HiF), were used. The diluent was distilled water, the aqueous fraction was nominally 10 % for all sources, and efficiency variation was achieved via addition of nitromethane. The ^3^H tracer sources were prepared with distilled water (no carrier) and counted the day after the ^18^F sources. *ε*_H-3_ ranged from 0.22 to 0.41. The ^18^F cocktails were dark adapted for < 100 minutes prior to counting. The UGAB cocktails were found to be unstable, showing a ≈ 2.5 % drop in recovered massic activity accompanied by a precipitous drop in QIP (where the QIP is tSIE, indicating increased quenching) over the first 10 h of counting. The UGAB data was therefore excluded from the final activity determination. Analysis of the HiF CNET data returned a massic activity that was 0.9 % lower than the LTAC-determined activity.

Unfortunately, all sources measured via TDCR were prepared with UGAB. Three LS sources were prepared with different amounts of ^18^F so that they had comparable count rates at the counting time. A matched blank was also counted for background subtraction. Sources were dark adapted for approximately 2 h after preparation, and all sources were counted over a span of 3.5 h. The TDCR data did not show trending with time or any other evidence of cocktail instability, but the massic activity calculated from these data was approximately 3.9 % lower than the value determined by LTAC and the sample-to-sample variability (≈ 0.6 %) was unusually high (it was ≈ 0.1 % in the experiments described in Secs. 3.9 and 3.10). Because of these issues, the TDCR-determined activity was rejected as erroneous. The discrepancy between TDCR and the other methods was the principal motivation for the experiments described in Secs. 3.9 and 3.10.

LTAC sources were dark adapted for only 3 h (ordinary LTAC protocols include a 12 h period of dark adapting), but the preparation of sources under incandescent light helped keep the background count rate very low (< 3.5 s^−1^) and stable with time. Decay-corrected intercept values for each source were constant over 9 half-lives, and the massic activities determined from 2 replicate LTAC sources agreed to 0.09(7) %. While the sources were prepared with UGAB, the simple LTAC model is not expected to suffer from cocktail instability the way CNET and TDCR do (see Sec. 2.3.2). The massic activity determined by LTAC had a combined uncertainty of 0.34 %.

Measurements on the 8-inch NaI(Tl) system were in excellent accord with the LTAC results, and achieved a combined uncertainty of 0.8 %.

IC measurements using previously determined calibration factors were in poor agreement with the LTAC-determined activity. Table 4 shows the results for all of the ICs used in this experiment. Using the 2001 CNET-based calibration factor, the VIC returned a massic activity 4.1 % lower than the LTAC-derived value. Using the LTAC activity to calculate a new K_VIC_ gives a value 0.2 % higher than the NPL value [96], meaning that the LTAC activity is in good accord with the CCRI(II)-K3.F-18 KCRV. Using DS = 472 (as determined in 1998 to 2001), the Capintec dose calibrators returned massic activities that were 3.6 % to 4.4 % lower than the LTAC-derived value (Table 4). Finally, using the 1982 K-value (found to return activities 5.1 % higher than the CNET-based VIC activity in 2008), the chamber “A” results agree with LTAC to within 0.5 %.

The IC results from this experiment indicated:

1. That the new LTAC-based standard was not consistent with the 1998 to 2006 NIST standardizations.
2. That the new LTAC-based standard was consistent with the chamber A calibration factor calculated in 1982.
3. That the new LTAC-based standard was consistent with the NPL value, and therefore with the KCRV from the CCRI(II)-K3.F-18 intercomparison.
4. That previous dose calibrator dial setting determinations performed at NIST needed to be reevaluated in light of the LTAC-based standard.

### 3.9 2012-II

Nähle and Kossert have enumerated the advantages of performing CNET and TDCR in concert [97]. The two techniques rely on the same model but differ in their sensitivity to different input parameters, so that accord can be taken as an indication that model-dependent biases are minimized. In June 2012, NIST performed a set of experiments in an effort to address the discrepancies between CNET and TDCR described in Sec. 3.8 [41].

LS efficiencies for CNET and TDCR were taken from the same calculations used in the preceding experiment. Hionic Fluor (HiF) was the scintillant. The source was [^18^F]FDG (from Cardinal Health, Baltimore, MD) and while no impurity measurements were made for this experiment, previous and subsequent (Sec. 3.10) experience with sources from the same supplier have identified no significant radionuclidic impurities. The diluent was distilled water, the aqueous fraction was nominally 10 % for all sources, and efficiency variation was achieved via addition of nitromethane. The HiF ^3^H tracer sources prepared in experiment 2012-I were recounted the day after the ^18^F sources, giving *ε*_H-3_ = 0.23 to 0.31. The ^18^F cocktails were dark adapted for ≈ 98 minutes prior to counting.

Two TDCR sources were prepared, the second containing approximately 3.5 times more FDG than the first. The cocktails were dark adapted for approximately 2 h prior to counting. Efficiency variation for the first source was achieved with gray filters. The second source was measured for > 15 h with one gray filter, establishing the linearity range for the TDCR. All counts were taken simultaneously with the MAC3 and FPGA-based electronics.

The massic activities recovered by the two techniques agreed to 0.13(66) %. Measurements on the VIC showed that the K_VIC_ determined with the 2012-II CNET activity was 4.0(8) % lower than the 2001 CNET K_VIC_; it was 0.08(67) % higher than the 2012-I LTAC K_VIC_. Using DS = 472, the CRC-12 dose calibrator returned an activity that was 2.5 % lower than the 2012-II CNET value. The CRC-12 dose calibrator returned the correct (2012-II CNET) activity with DS = 452, in good agreement with a determination made using the 2012 LTAC activity (Sec. 3.8).

**Fig. 4:**
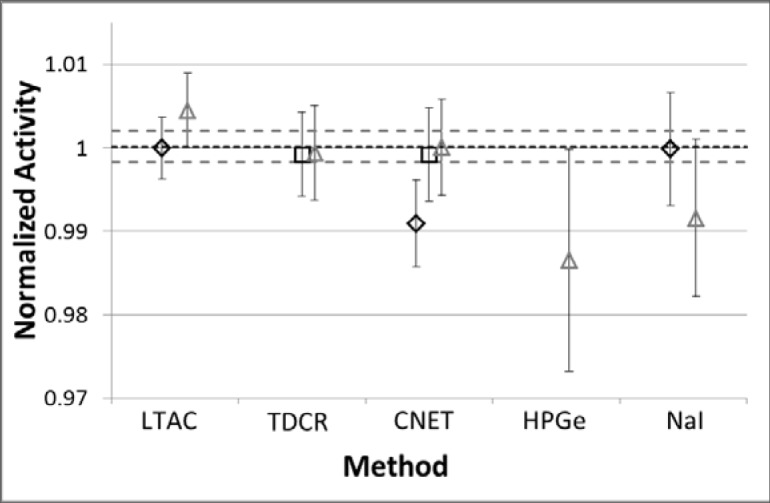
Comparison of NIST ^18^F standardization results from 2012 to 2013 [41]. All results are normalized (via K_VIC_ links) to the 2012 LTAC determination. The different symbols correspond to the three different experiments: open diamonds – 2012-I; open squares – 2012-II; open triangles – 2013. The uncertainty bars represent combined standard uncertainties (*k* = 1). The dotted lines represent the KCRV from the CCRI(II)-K3.F-18 comparison [25] and its standard uncertainty. TDCR data from 2012-I is not represented in this figure (see Sec. 3.8 for details).

In summary, the IC measurements established that the new CNET and TDCR determinations were in excellent accord with LTAC (see Fig. 4).

### 3.10 2013

Because the new LTAC-based standard differed so substantially from the NIST standard that was found to be consistent across three earlier primary standardizations (Secs. 3.4 to 3.6), NIST performed another round of measurements involving CNET, TDCR, and LTAC. IC measurements were performed to provide links with past experiments. Activity measurements were also performed with the 8-inch NaI(Tl) system. HPGe measurements were performed to assess impurities and determine the ^18^F activity.

LS efficiencies for CNET and TDCR were taken from the same calculations used in the 2012 experiments. Hionic Fluor was the scintillant. The source was [^18^F]FDG (from PETNET, Baltimore, MD), the diluent was distilled water, the aqueous fraction was nominally 10 % for all sources, and efficiency variation was achieved via addition of nitromethane. The ^3^H tracer sources were prepared with distilled water (no carrier) and *ε*_H-3_ ranged from 0.25 to 0.34. The tritium sources were counted after the ^18^F sources. The ^18^F cocktails were dark adapted for 3 h prior to counting.

As part of this experiment, one LS source was prepared with extremely high (≈ 1.8 · 10^8^ Bq) activity at the time of preparation and allowed to decay prior to measurement. The goal was to measure any possible degradation of the cocktail due to high dose prior to measurement. Since the time between cocktail preparation and measurement varied widely over the many NIST standardizations, this was thought to be a potentially important variable. Analysis of this source via CNET returned a massic activity 0.3(7) % higher than the value determined with the low-level sources. Furthermore, fluorescence spectroscopy showed no diminution in the fluorescence efficiency of the decayed source. This source also provided an excellent measure of the ^3^H impurity (2·10^−4^ % at the reference time) of the [^18^F]FDG solution.

Three TDCR sources were prepared, the third containing approximately 30 times more FDG than the first two. The cocktails were dark adapted for approximately 2 h prior to counting. Efficiency variation for the first two sources was achieved with gray filters. The third source was counted for > 11 h with one gray filter, providing 61 data points within the instrument’s linearity range. All counts were taken simultaneously with the MAC3 and FPGA-based electronics.

Two LTAC sources, linked by a dilution factor of ≈ 270, were measured by the same protocols described in Sec. 3.8. The massic activities recovered for the two sources agreed to 0.03(11) %, confirming the gravimetric dilution factor. The K_VIC_ determined using the 2012 LTAC activity was 0.45(58) % lower than that from 2013.

The activity determined via the 8-inch NaI system was 1.3(1.1) % lower than the 2013 LTAC value, and, using the K_VIC_ link (as in Ref. [41]), was 0.9(1.0) % lower than the 2012 LTAC value. The 2013 NaI value was 0.9(1.2) % higher than the 2012 NaI value. One reason for the 0.9 % difference may be that the 2012 NaI measurement was on an LTAC LS hemisphere, whereas the 2013 measurement was on a NIST standard 5 mL ampoule.

HPGe measurements revealed no gamma-emitting impurities. The activity determined via HPGe had a combined uncertainty of 1.3 %, and was 1.8(1.4) % lower than the 2013 LTAC value; using the K_VIC_ link, it was shown to be 1.4(1.4) % lower than the 2012 LTAC value.

CNET and TDCR agreed with each other to 0.1(8) % and with LTAC to 0.4(7) % and 0.5(7) %, respectively. Using the K_VIC_ link, it was established that the 2013 CNET and TDCR values both agreed with the 2012 LTAC value to < 0.1(7) %.

Ionization chamber measurements were performed on the CRC-12 and VIC, yielding chamber responses in pA·MBq^−1^ that were, respectively, 0.8(1.5) % and 0.4(3.0) % higher than those determined in the 2012-I experiment.

In summary, the 2013 experiments were in excellent accord with the 2012 studies (Fig. 4). The 2012 LTAC value was adopted as the new primary standard upon which secondary standards (IC calibration factors) were based [41].

## 4. Discussion

### 4.1 Linking the Standards

Figure 2 shows the results of all of the NIST standardizations, normalized to the 2012 LTAC-based ^18^F standard, and how they are linked.

For most ionization chambers (IC), links were established from the relative difference between the LSC-determined activity and the IC-determined activity using a given calibration factor. For example, a measurement taken on NIST chamber “A” as part of the 1982-II experiment returned an activity 0.3(9) % higher than the CNET-derived value. The corresponding point (∆^°^_1982−II_) in Fig 2 was normalized according to

$$\Delta_{1982-\mathrm{II}}^{\circ }=\Delta_{1982-\mathrm{II}}-\Delta_{\mathrm{LTAC}}+1$$ (5)

where ∆_1982−II_ is the relative difference between the CNET result and the chamber “A” result (−0.0027) and ∆_*LTAC*_ is the relative difference between the 2012 LTAC result and the chamber “A” result (0.0047). The link is possible because the same calibration factor (with appropriate corrections) was applied to both measurements.

The VIC linkages were treated only slightly differently, with the K_VIC_ (in units of pA·MBq^−1^) serving as the link. The CNET-derived activity from the 2001 experiment was stored as a K_VIC_ which was then compared to the results in later experiments. Again, results were normalized to the 2012 LTAC result for ease of comparison.

For linkages involving Capintec chambers, the strongest links are those established with the same chamber and dial setting, at the same facility and in the same configuration; as described in the caption to Fig. 2, such ideal linkages were not always available, making some linkages clearly more robust than others. Perhaps the most suspect link is for the 1982-I experiment, where the comparison relies on a pair of Capintec dose calibrators that were in use at NIH at the time of the experiments. This particular linkage is thus potentially confounded by a great number of variables outside the control of NIST scientists, such as the manufacturer’s consistency (in terms of, for example, response linearity and calibration factors) between different chamber models over a span of years and the QC procedures in place at NIH.

### 4.2 The LTAC-Based ^18^F Standard

The 2012 LTAC-based standard is in good agreement (−0.21 %) with the results from NPL [96]. From degrees of equivalence and the KCRV, we calculate that the NPL-reported value for the CCRI(II)-K3.F-18 comparison was 0.20 % higher than the KCRV and had a standard uncertainty of 0.39 %. Thus, although the links are indirect, the new NIST ^18^F standard appears to be in accord with the International Reference System (SIR) reference value (KCRV, Fig. 4).

The resolution of the discrepancy between NIST and the SIR is encouraging. However, even if we had never participated in the CCRI(II)-K3.F-18 comparison, we would be changing the NIST standard. The new NIST ^18^F standard is based on a more robust technique (LTAC) than the previous standard. It is based on a series of experiments using a variety of measurement techniques, including the technique (CNET) that was the basis for the previous standard. As reported by Fitzgerald *et al*. [41], agreement between the various techniques is very good (Fig. 4).

### 4.3 1998–2006 Consistency

It is troubling that repeated primary standardizations at NIST over the course of a decade produced consistently biased results. The principal reason for this review has been to collect the relevant data that might allow us to attribute this bias to a specific cause. And then to avoid that cause in the future.

We have considered many possible mechanisms for the observed bias, and we have eliminated many of them.

#### 4.3.1 Model

Regular users of the CNET technique will be aware that model-dependences can pose serious problems. The very high counting efficiency of ^18^F would be expected to result in minimal model-dependence, and in fact our review of the data revealed that all efficiency codes employed in our LSC standardizations gave similar results. The 2001 experiments utilized the CN2000 code, a version of which was employed in all subsequent experiments. In the 2012 and 2013 studies, the equivalence of CN2004 and MICELLE2 efficiency calculations was demonstrated. In these models, dependence on *kB* is typically less than 0.01 %, and ^18^F efficiency varies by only 0.25 % over the range of *ε*_H-3_ = 0.2 to 0.5. Model-dependences cannot explain a 4 % discrepancy.

#### 4.3.2 LS Counter

Since the standardizations were performed at several different sites with several different LS counters, it is logical to question whether instrumental differences might have contributed to the observed bias. Consistent results in the 1998 to 2006 experiments and the 2012 to 2013 experiments were achieved with several different LS counters and with overlapping ranges of count rates (Table 3). The 2001 experiment was performed at NIST on the Packard Tri-Carb A2500 TR. This same counter was used in the 2012 and 2013 experiments, providing strong evidence against a specific instrumental bias.

#### 4.3.3 Source Type/Impurities

As discussed in detail in Sec. 3.4, an improper accounting of radionuclidic impurities can be particularly problematic in assays of short-lived nuclides like ^18^F. The ^18^F sources used in the various NIST standardizations took several different forms (target water, fluoride, or FDG), and thus had different levels of impurities. In some experiments, no impurity measurements were made. Our reanalysis of the 1998 experiment suggests a previously unaccounted ^3^H impurity. However, the reanalysis does not significantly change the activity. In the 2006 experiment, LS measurements showed no long-lived β-emitting impurities (i.e., ^3^H) and HPGe measurements indicated no γ-emitting impurities. The 2012 and 2013 experiments also took careful account of β- and γ-emitting impurities, so the discrepancy with the 2006 results cannot be attributed to impurity effects.

#### 4.3.4 Scintillant Effects

Scintillant selection and formulation influence counting efficiencies in several ways. Each specific commercial scintillant has a different intrinsic efficiency, a different optimal aqueous fraction, a different acid/base tolerance, and different optical properties such as quench function.

Records indicate the specific usage of six different scintillants. In three experiments, more than one scintillant was used; it is typically advisable to use more than one scintillant in an experiment in order to improve the probability of finding a stable cocktail formulation and to identify and hopefully explain and eliminate any dependence of the result on cocktail selection. In two experiments, there is no record of which scintillant(s) were used. All of the 2012 and 2013 experiments used Hionic Fluor (HiF).

Only one scintillant, Ultima Gold AB (UGAB), bridges the two discrepant sets of results (1998 to 2006 versus 2012 to 2013); the 2012 formulation was found to be unstable, while the 1998 formulation was not. It is possible that the long delay (12 h) between cocktail preparation and measurement obscured an initial instability in the ^18^F cocktails in the 1998 experiment. However, it is also possible that the FDG used in the 1998 experiment produced a more stable formulation than the target water used in 2012. Finally, since manufacturers periodically change scintillant compositions, there is no guarantee of a valid comparison between batches of UGAB from 1998 and 2012.

The 2006 experiment looked explicitly for cocktail instability in two scintillants and found none, indicating that any decrease in counting efficiency would have had to have occurred in the relatively brief period (approximately 185 minutes, according to the logs) between source preparation and counting. However, the low aqueous fractions used in the cocktails in 2006 raise the open question of whether cocktail instability might have been an undetected problem.

In light of the available evidence, it is impossible to say whether cocktail instability contributed substantially to the discrepancy between 1998 to 2006 and 2012 to 2013 experiments. The 3.9 % difference between the UGAB-based TDCR results and the LTAC and HiF-based CN results (Experiment 2012-I) is comparable in magnitude to the observed discrepancy in the NIST standards. It is therefore tempting to claim that an explanation has been found. However, we do not feel that the evidence is sufficient to aver such a claim. We hope in the future to address this problem experimentally.

Finally, we determined via LS and fluorescence spectroscopy that even higher than normal dose does not produce radiation damage to HiF LS cocktails that appreciably affects the fluorescence efficiency.

## 5. Conclusions

The recently reported LTAC-based primary radioactivity standard for ^18^F [41] represents a change from the consistent NIST values from 1998 to 2006. A review of ten NIST standardizations performed over a span of 31 years has demonstrated that the experimental protocols have differed substantially. The different procedures arise from changes in personnel, improvements in metrological techniques, and sometimes the locale and goal of the study. All standardizations included liquid scintillation counting (LSC)-based measurements. Early LSC experiments were not explicitly CIEMAT/NIST efficiency tracing (CNET) studies, but utilized techniques that were CNET precursors. The LSC results were linked through ionization chamber (IC) measurements.

An analysis of the linkages underscored the consistency of the NIST ^18^F primary standard from 1998 to 2006. This standard was disseminated as secondary standards for dose calibrators [38,39,87,88] and was consistent with the NIST submission to the CCRI(II)-K3.F-18 comparison [25,35]. NIST differed from the KCRV in this comparison by approximately 3.8 %.

A series of experiments performed in 2012 and 2013 are inconsistent with this earlier standard. In these experiments, CNET was complemented by triple-to-double coincidence ratio (TDCR) LSC and live-timed anticoincidence counting (LTAC). Confirmatory measurements were also performed with two γ-counting methods. LTAC is less model-dependent than CNET and TDCR, and is therefore considered to be a more robust primary method for this case. The 2012 and 2013 experiments achieved accord between all three of these techniques, arriving at a new primary activity standard for ^18^F. The LTAC-based standard differs from the earlier standard by 4.0 %. Though linked only indirectly, the LTAC-based standard is in much better accord with the CCRI(II)-K3.F-18 KCRV.

This review has enumerated the sources of experimental bias considered as possible explanations of the 4.0 % discrepancy. We cannot yet definitively attribute the discrepancy to a specific cause, but investigations—especially surrounding the effects of LS cocktail instability—are ongoing. The discord discovered in the NIST standard for ^18^F underscores the importance (when practicable) of: 1) including multiple primary techniques in a standardization; 2) including and carefully considering secondary links to previous studies; and 3) checking and correcting for impurities, including non-γ-emitting impurities.

The change in the ^18^F standard will have serious implications for calibrations in nuclear medicine. We have already reported on revised NIST determinations for some dose calibrator dial settings [41]. We are reviewing the implications of the new standard for the ^68^Ge surrogate calibration method described in [87]. We have discussed the new standard with some stakeholders and we continue to disseminate our results as appropriate.

## Figures and Tables

**Table 1:** Standardizations of ^18^F by national metrology institutions (NMIs). A list of NMI acronyms can be found online [98].

Laboratory	Primary Method(s)	Uncertainty (*k* = 1), %	Reference(s)
ANSTO	4πβ-γ coincidence counting efficiency-tracing extrapolation method	1.4	[25,29]
BEV	pressurised ionization chamber	1.0	[22]
CIEMAT	pressurized IC; 4πβ(PPC)-γ coincidence; CNET	0.48 to 0.63	[24,27,31,32]
CMI-IIR	4πβ(PC)-γ coincidence	0.44	[25]
CNEA	4πβ(PC)-γ coincidence	0.57	[31]
ENEA	CNET	0.51	[26]
INER	4πβ(PC)-γ coincidence	0.51	[25]
IPEN	4πβ(PC)-γ coincidence	0.58	[23]
IRA	pressurised ionization chamber; 4πβ-4πγ coincidence	0.30 to 0.55	[22,30]
LNE-LNHB	TDCR-LSC	1.0	[22,31]
NIRH	IC measurement	1.1	[23]
NIST	CNET; pressurized IC; TDCR-LSC; LTAC(LS)	0.44 to 0.70	[25,37–39,41,79] *and herein.*
NMIJ	4π(β+γ); 4πβ(PC)−γ; 4πβ-4πγ coincidence	0.2 to 0.5	[36]
NPL	4πβ(PC)-γ coincidence	0.24	[23,28,34]
PTB	pressurized IC; 4πβ(PC)-γ coincidence; CNET	0.32	[25,33]

**Table 2:** Summary of the techniques employed in NIST standardizations of ^18^F from 1982 to 2013.

Method	1982-I	1982-II	1992	1998	2001	2006	2008	2012-I	2012-II	2013
LSC / CNET	X	X	X	X	X	X		X	X	X
TDCR								X	X	X
LTAC								X		X
8″ NaI system								X		X
Gamma Spec (HPGE or Ge(Li))	X	X	X					X		X
Ionization Chambers	X	X	X	X	X	X	X	X	X	X

chamber “A”	X*	X	X				X	X		
AutoIC								X		
VIC					X		X	X	X	X
CRC-12			X	X	X		X	X	X	X
CRC-15R						X		X		
CRC-35R				X				X		
CRC-1.8 atm								X		X

* The *K*-value used in this experiment (in units of positrons per second rather than Bq) was not directly comparable to the value used in later studies.

**Table 3:** Summary of liquid scintillation counting experiments. The commercial LS counters are referred to as: P3320 – NIST Packard 3320; PTC – NIST Packard TriCarb A2500; P-ORNL – Oak Ridge National Laboratories Packard TriCarb 2900TR; B – NIST Beckman LS7800; W – Wallac 1414 Winspectral. Commercial scintillants are referred to as: PF – PicoFluor; IG – Instagel; UGAB – Ultima Gold AB; RS – Ready Safe; OF – OptiFluor; HiF – Hionic Fluor. F-18 samples were in the form of potassium fluoride (KF), sodium fluoride (NaF), or Fluoro-2-Deoxy-D-Glucose (FDG). The time elapsed between source preparation (*t*_prep_) and counting (*t*_count_) is given as *t*_count_ – *t*_prep_.

	LS Counter(s)	Scintillant(s)	^18^F Sample	Efficiency Model	ε_H-3_ Range	Count rate/cps	*t*_count_ – *t*_prep_
	
1982-I	P3320	unknown	KF	100 %*		≈ 960	< 2 h
1982-II	P3320	PF, IG	FDG	AGM**	0.36 to 0.43	480 to 1650	< 3 h
1992	PTC / B	unknown		99.96 %***		900 to 1250	< 2 h
1998	B	UGAB	FDG	EFFY	0.4 to 0.41	850 to 6500	≈ 12 h
2001	PTC / B		NaF	EFFY+EMI, CN2000	≈ 0.4	800 to 3300	< 2 h, ≈ 12 h
2006	P-ORNL	RS, OF	FDG	CN2004	0.40 to 0.57	1201 to 7600	3.08 h
2012-I	PTC	UGAB, HF	NaF	CN2004, MICELLE2	0.22 to 0.41	300 to 8800	1.67 h
2012-II	PTC	HF	FDG	CN2004, MICELLE2	0.23 to 0.31	300 to 5770	1.63 h
2013	PTC / W	HF	FDG	CN2004, MICELLE2	0.25 to 0.34	600 to 4·10^6^	≈ 3 h

* A positron branching ratio of 0.969 was used with the assumption of 100 % counting efficiency for positrons.

** Efficiency calculations by Augustín Grau Malonda gave a *ε*_F-18_ range of 0.951 to 0.957, but a positron branching ratio of 0.969 with assumed 100 % counting efficiency was used in the final data analyses.

*** A positron branching ratio of 0.969 was used with the assumption of 99.96 % counting efficiency for positrons.

**Table 4:** Comparison of ionization chamber (IC) calibration factors (*CF*s) determined with the pre-2012 and LTAC-based standards for ^18^F activity. The chamber “A” K-value is given in units of Bq, and the pre-2012 value was based on the 1982 (^22^Na-based) theoretical determination. K_VIC_ is given in pA·MBq^−1^, as is the *CF* for the Capintec CRC-12 in “current mode”. For each Capintec dose calibrator, *CF*s are reported in “dial setting” (DS) units. The percent difference between the measured activities using the two *CF*s is given as *Δ*.

IC	*CF*_pre-2012_		*CF*_2012_		*Δ*/%
chamber “A”	5.28E+07	Bq	5.31E+07	Bq	0.47
VIC	10.80	pA·MBq^−1^	10.36	pA·MBq^−1^	4.1
CRC-12	8.647	pA·MBq^−1^	8.276	pA·MBq^−1^	4.5
CRC-12	474–477		450		4.4
CRC-15R	472		449		4.2
CRC-35R	470–472		450		3.6
CRC-25PET	455*		455		–
CRC-1.8 atm	–		486		–

* For the CRC-25PET, *CF*_pre-2012_ is based on the manufacturer’s preset ^18^F DS, and not on a NIST determination.

## Footnotes

1 Certain commercial equipment, instruments, and materials are identified in this paper to foster understanding. Such identification does not imply recommendation or endorsement by National Institute of Standards and Technology, nor does it imply that the materials or equipment are necessarily the best available for the purpose.
